# Burkitt’s lymphoma with placental invasion diagnosed at cesarean delivery: a case report

**DOI:** 10.1186/s13256-017-1548-0

**Published:** 2018-02-08

**Authors:** Cielo Gnecco, S.J. Carlan, Jeannie McWhorter, Li Ge, Daniel Sanchez, Mario Madruga

**Affiliations:** 1 Department of Pathology, Orlando Regional Healthcare, 1401 Lucerne Terrace, 2nd floor, Orlando, FL 32806 USA; 2 Department of Internal Medicine, Orlando Regional Healthcare, 1401 Lucerne Terrace, 2nd floor, Orlando, FL 32806 USA; 3 Department of Obstetrics and Gynecology, Orlando Regional Healthcare, Orlando, FL USA

**Keywords:** Pregnancy, Placenta, Burkitt’s lymphoma, Cesarean delivery, Prematurity

## Abstract

**Background:**

Burkitt’s lymphoma is a highly aggressive B cell non-Hodgkin lymphoma subtype. Its occurrence in pregnancy is rare and often results in a delayed diagnosis. The treatment plan and prognosis depend on a number of variables including the stage at diagnosis.

**Case presentation:**

A 32 weeks pregnant, 34-year-old white woman presented with weeks of complaints that were similar to typical pregnancy symptoms. Laboratory and ultrasound findings suggested a pathologic process and during the workup non-reassuring fetal surveillance resulted in an emergency cesarean delivery. Biopsies were obtained that confirmed Burkitt’s lymphoma. Placental histology revealed microscopic involvement.

**Conclusions:**

The placenta should be inspected for microscopic disease if Burkitt’s lymphoma is suspected, even if a vaginal delivery occurs and the placenta is ordinarily discarded. Repetitive somatic complaints during pregnancy should not be assumed to be secondary to the normal symptoms of pregnancy.

## Background

Burkitt’s lymphoma (BL) is an aggressive subtype of non-Hodgkin lymphoma (NHL) that is characterized by the translocation and deregulation of the *c-myc* gene on chromosome 8 [1]. It frequently presents at extranodal sites [2] and is extremely rare during pregnancy [3]. BL is the fastest known growing human tumor with a cell doubling time of 24 to 48 hours. In addition, it is the first human tumor to be associated with a virus [4]. The diagnosis can be delayed in pregnancy which may influence the stage of cancer and, therefore, the treatment plan and prognosis [5]. Metastasis of BL to reproductive organs during pregnancy can occur [6]; however, metastasis to the placenta has not been reported. We report a case of BL in a 32-week pregnancy with ascites, macroscopic peritoneal and omental implants, and placental metastasis. This case shows that metastasis to the placenta can occur and when BL is suspected the placenta should be sent for histologic evaluation rather than discarded.

## Case presentation

A 34-year-old unemployed white woman, gravida (G) 5 para (P) 1223, at 32.6 weeks of gestation presented to her primary care physician with worsening back and rib pain, right lower extremity edema, and abdominal distension for several weeks. She reported that she had been evaluated several times for these symptoms and those of anorexia, nausea, and vomiting. She had been unable to tolerate oral intake and had been noticing increased edema and erythema in the periumbilical and suprapubic areas of her abdomen. She was diagnosed as having cellulitis and given orally administered cephalexin without improvement of her symptoms. She presented to our hospital with worsening of her symptoms and was found to have ascites and transaminitis. On arrival her blood pressure was 114/50, pulse 78, respirations 18, pulse 78, and temperature 98.3 °F (36.83 °C). She denied a history of hepatitis, sexually transmitted diseases, or other significant illnesses. She did not use tobacco or drugs, and had not traveled outside the USA recently. She had never had an abnormal Papanicolaou test. Her family history was only positive for her mother and maternal grandmother having splenectomies for an unknown reason. Her past surgical history revealed a laparoscopic salpingectomy, two previous cesarean deliveries, and a splenectomy for a spleen rupture secondary to mononucleosis. Her past medical and family histories were otherwise unremarkable. She had been on no medications other than orally administered cephalexin.

**Fig. 1:**
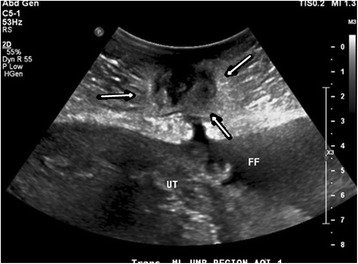
Ultrasound of the abdomen showing free fluid with echoes in the ascitic compartment representing clinically insignificant sound reverberation and floating debris outside the uterine wall. Periumbilical abdomen demonstrates an area of abnormal heterogeneous soft tissue (*arrows*) with an area of complexity posterior to the umbilicus 5.1 × 4.6 × 3.1 cm seemingly with some underlying continuity with the peritoneal cavity. No identifiable abscess or echo suspicious for solid tumor metastasis. *FF* free fluid, *UT* shows the uterine wall

On physical examination, her abdomen was found to be distended, with a well-circumscribed region of erythema approximately 8 cm in diameter surrounding the umbilicus. Her neurologic examination was intact and her psychiatric examination was negative. A sonogram of her abdomen showed moderate diffuse ascites and an abnormal complexity at the periumbilical abdomen, probably continuous with the peritoneal cavity (Fig. 1). A right upper quadrant sonogram showed that her liver had normal echogenicity, measuring around 15.9 cm in length with no focal lesions. A laboratory workup was significant for the following results: white blood cell count of 11,500/mcL, hemoglobin of 11.7 g/dL, aspartate aminotransferase of 129 U/L, alanine aminotransferase of 52 U/L, and lactic acid of 5.8 mmol/L. Total bilirubin was 1.1 mg/dL and alkaline phosphatase was 128 U/L. Cancer antigen 19-9 and cancer antigen 125 were elevated at 196.7 U/mL and 145 U/mL, respectively. Serological tests for human immunovirus, hepatitis B, and hepatitis C were negative. An echocardiogram showed an ejection fraction of 65 to 69%. She suddenly developed non-reassuring fetal surveillance requiring urgent delivery. At the time of cesarean delivery, approximately 8 liters of ascitic fluid were obtained and a sample was sent for cytology. She delivered a 1650 gram premature baby girl with an umbilical cord pH of 7.01 and Apgar scores of 2 and 8. A 343 gram morphologically normal placenta was manually removed and sent to pathology. Biopsies were obtained of her omentum which was thickened, inflamed, and adhered to her anterior abdominal wall. Her anterior abdominal wall was palpated and there did not appear to be a collection of fluid or abscess within the wall at the umbilicus or at the cellulitis site. Her peritoneum was also noted to be thickened and inflamed and was also biopsied. Her liver was palpated and there were multiple small implantations felt along the anterior surface.

**Fig. 2:**
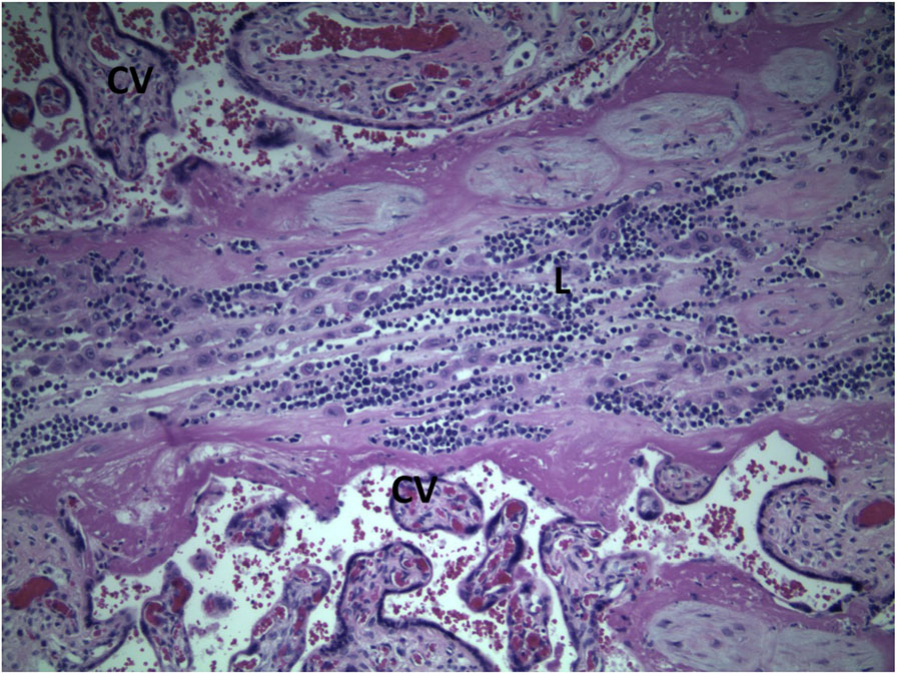
Lymphoma cells infiltrate decidua (*middle*) in between chorionic villi (*top* and *bottom*). Hematoxylin-eosin stains, × 100. *CV* chorionic villi, *L* lymphoma cells

**Fig. 3:**
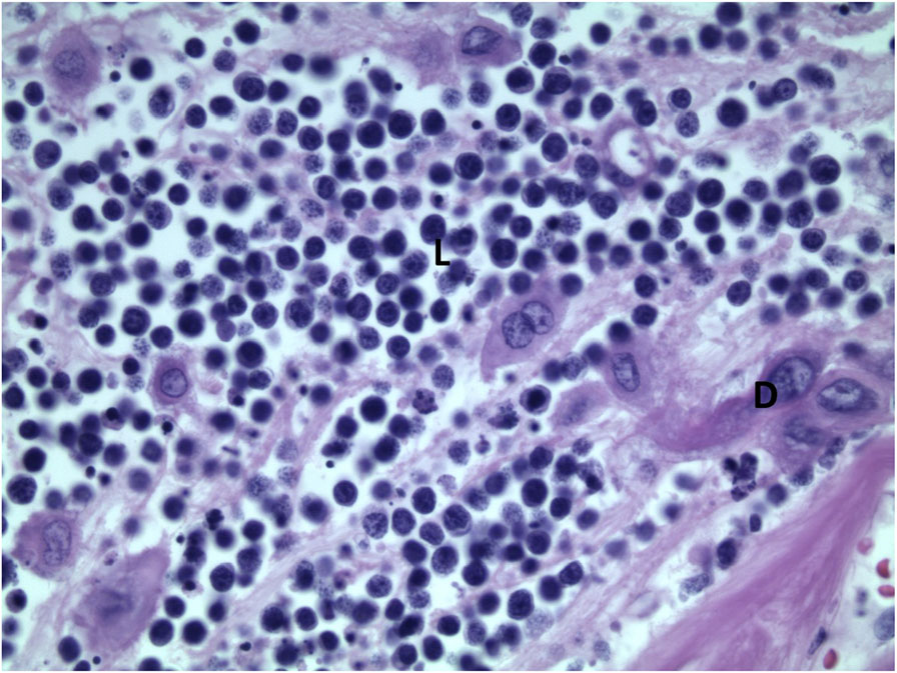
High magnification of placenta shows monotonous population of medium-sized lymphoma cells infiltrates between scattered larger decidual cells. Hematoxylin-eosin stains, × 400. *D* decidual cells, *L* lymphoma cells

**Fig. 4:**
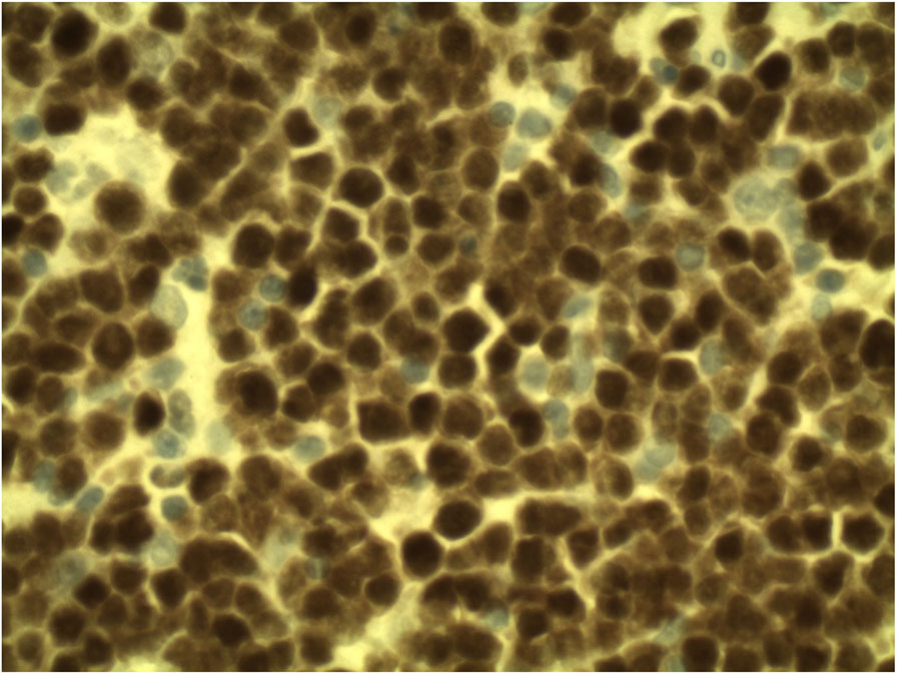
Lymphoma cells are strongly positive for PAX5 immunochemical staining (×600)

**Fig. 5:**
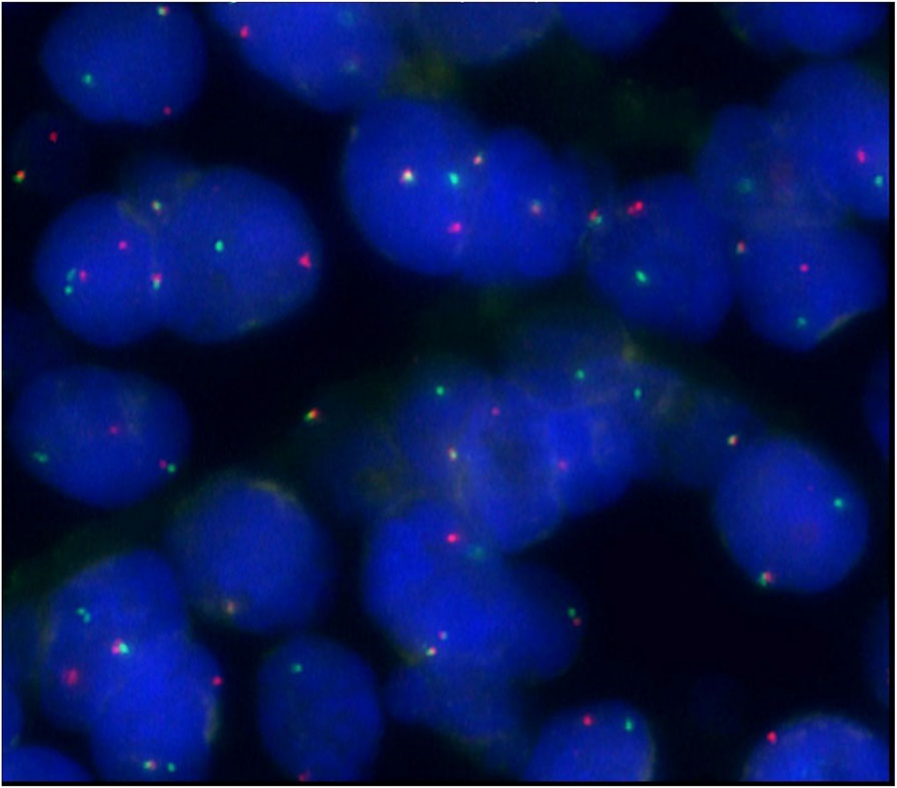
Fluorescence *in situ* hybridization analysis is positive for *MYC* gene rearrangement; the abnormal signal pattern (1 red, 1 green, 1 fusion; negative < 5%) in *MYC* was observed in 78% of the analyzed nuclei

Postoperatively, she developed worsening ascites and a paracentesis yielded and an additional 5.5 L of yellow ascitic fluid. After the second paracentesis, she was diagnosed as having an ileus and a nasogastric (NG) tube had to be placed. Pathology reports of omental biopsies taken during delivery were consistent with the diagnosis of BL. Pathology of the placenta indicated microinvasive involvement by BL (Figs. 2 and 3). Immunohistochemical studies showed positivity for cellular myc oncogene (cMYC), cluster of differentiation (CD) 20, and paired-box 8 (PAX5; Fig. 4). The tumor was also positive for B-cell CLL/lymphoma 6 (Bcl6), CD10, and showed greater than 95% reactivity for Kiel 67 (Ki67). Stains for CD3, B-cell lymphoma 2 (bcl2), CD5, and CD34 were negative and there was also negativity for the 14:18 translocation. A lumbar puncture was performed and cerebrospinal fluid was negative for malignancy. A computed tomography scan showed no lung involvement. Per Ann Arbor staging criteria [7], our patient was diagnosed as having stage IV BL and she was started on unfractionated cyclophosphamide, dexamethasone 40 mg intravenously for 5 days, and rituximab 375 mg/m^2^. The plan was to switch this regimen to R-EPOCH (rituximab, etoposide, prednisone, vincristine, cyclophosphamide, and doxorubicin) once clinically stable. After initiation of chemotherapy, she developed acute renal failure and required emergency hemodialysis. She developed flash pulmonary edema and required intubation and then developed septicemia and pneumonia caused by *Citrobacter braakii*. She eventually improved after 4 days of treatment and medical oncology recommended initiation with R-EPOCH for a total of six cycles. She was discharged home with instructions to return for first cycle of chemotherapy. Prior to discharge she was started on allopurinol for treatment of hyperuricemia due to ongoing tumor lysis syndrome. Her premature baby girl remained in our neonatal intensive care unit for 19 days after delivery. Due to the presence of micro-infiltrates of BL in placenta, hematology was consulted to rule out malignancy in newborn. A chest X-ray and ultrasound of the baby’s abdomen were normal. Fluorescence *in situ* hybridization analysis showed no presence of *c-myc* translocation in the baby girl (Fig. 5). A peripheral smear was negative and laboratory studies showed no abnormalities. The baby girl was discharged home with disposition to follow up with hematology out-patients for routine laboratory studies; she has had no health conditions other than the usual maladies of babies. The mother, however, has been admitted four times since her discharge for both chemotherapy encounters and symptoms of peritoneal carcinomatosis, sepsis, and gastrointestinal complaints. At 5-month post-diagnosis she was lost to follow up by our team and is presumed to have found care in her own region of the state.

## Discussion

This case is important because it is the first report of invasion of BL to the products of conception. Our patient was seen and treated multiple times for what were thought to be either normal complaints of pregnancy or cellulitis but this case illustrates the importance of timely consultation and imaging when the patient does not improve. Finally, the placenta usually is just discarded after a vaginal delivery; if there is any clinical suspicion of BL the products should be sent for histologic analysis.

There are two unusual features to this case. First, this case of BL occurs during pregnancy which can affect diagnosis, staging, and treatment. Pregnancy is a condition that is notable for delayed diagnosis of multiple conditions including some malignancies. The normal symptoms of pregnancy and the reluctance of clinicians to pursue clinical investigations during pregnancy are operative variables in the delay [5]. This patient had a measureable delay in diagnosis despite repeated complaints probably because the complaints are commonplace in normal pregnancy. The only difference in this patient is that they were the same complaints repeated multiple times. Nonetheless, BL is a highly aggressive germinal B cell lymphoma and therefore a delay may compromise maternal chances of cure or survival. When she finally presented to the tertiary referral center her laboratory and ultrasound findings documented a pathologic process. The cesarean delivery for fetal distress documented the reason for the clinical presentation when implants were found. The non-reassuring fetal heart rate pattern represented uteroplacental insufficiency and fetal hypoxia most likely secondary to the fluid shifts involving the ascites and third-spacing which acutely reduced the available uterine blood flow. Of note is that the baby girl was severely acidotic at birth. Even without the emergency cesarean delivery and concurrent biopsies, however, the diagnosis would have been made soon considering her abnormal laboratory results and ascites. This case is consistent with reports that acknowledge that delayed diagnosis in pregnancy is a serious threat that is even more significant since these tumors are potentially curable in the early stages [5].

The second unusual element to this case is the placental involvement. The incidence of malignancy in pregnancy ranges from 0.07 to 0.1% [8] and lymphoma is the fourth most common type of cancer resulting in one in 6000 pregnancies complicated by a new diagnosis of lymphoma [5]. Horowitz *et al*. [6] reported on 121 patients from 1967 to 2011 with NHL during pregnancy and 47% were highly aggressive Burkitt’s or immunoblastic subtypes. None of the cases of BL had placental involvement in their report. We performed a Medline search of the English language literature from 1 January 1966 to 31 December 2016, using the keywords “Burkitt’s lymphoma,” “metastasis,” “placenta,” and “pregnancy.” We could find no cases of BL metastasized to the placenta confirmed by histology. Our case appears to be the first and is notable because if BL does grossly invade the placenta and underlying decidual attachment, obstetric image interpretation may be misleading and decisions regarding delivery timing and method may be influenced. Moreover, our patient’s findings suggest that the placenta can become a site of metastasis in advanced cases and that if BL is suspected and the patient delivers vaginally then the placenta should be sent for histologic analysis in pathology. Ordinarily the placenta is simply discarded after a vaginal delivery but this case demonstrates that the placenta is also a reproductive organ that possibly can be involved as a metastatic site. Whether placental involvement, however, is a variable in treatment is undetermined especially considering the placenta is removed and discarded at birth. It may be that the fact the placenta is involved suggests that the cancer is particularly virulent and closer post-treatment surveillance is indicated. In addition, one other element to placental involvement is the possibility of metastasis to the baby girl. Since 1986, however, there have been only six documented cases of placental involvement by NHL and none had BL [9]. The initial workup of the child was negative but a surveillance screening schedule was recommended. Metastasis across the placenta is rare but the placenta must necessarily be involved for the child to become afflicted. Treatment generally consists of multi-agent chemotherapeutic agents depending on the patient’s age and initial performance status, as well as tumor specifics such as histological subtype, grade, and stage [5]. Possibly, placental and membrane involvement should be considered, as well, even though these tissues are removed at childbirth in most cases.

## Conclusions

In conclusion, the symptoms of pregnancy can result in a delayed diagnosis of BL which can influence the staging and outcome. The response of this cancer to treatment can be favorable when implemented early, thus, when complaints consistent with normal pregnancy symptoms are repetitive a workup is prudent. BL can metastasize to the placenta in pregnancy and histologic examination should be carried out if BL is considered a diagnosis. The implications of placental metastasis on staging and prognosis are undetermined but probably suggest an aggressive or late stage cancer.

## Abbreviations

Bcl2: B-cell lymphoma 2
Bcl6: B-cell CLL/lymphoma 6
BL: Burkitt’s lymphoma
CD: Cluster of differentiation
cMYC: Cellular myc oncogene
G: Gravida
Ki67: Kiel 67 (number of the original clone in the 96-well plate)
NHL: Non-Hodgkin lymphoma
P: Para
PAX5: Paired-box 5
R-EPOCH: Rituximab, etoposide, prednisone, vincristine, cyclophosphamide, doxorubicin
